# A Fucosylated Lactose-Presenting Tetravalent Glycocluster Acting as a Mutual Ligand of *Pseudomonas aeruginosa* Lectins A (PA-IL) and B (PA-IIL)—Synthesis and Interaction Studies

**DOI:** 10.3390/ijms232416194

**Published:** 2022-12-19

**Authors:** Magdolna Csávás, László Kalmár, Petronella Szőke, László Bence Farkas, Bálint Bécsi, Zoltán Kónya, János Kerékgyártó, Anikó Borbás, Ferenc Erdődi, Katalin E. Kövér

**Affiliations:** 1 Research Group for Molecular Recognition and Interaction, Eötvös Loránd Research Network, University of Debrecen, Egyetem tér 1, H-4032 Debrecen, Hungary; 2 Department of Ecology, University of Debrecen, Egyetem tér 1, H-4032 Debrecen, Hungary; 3 Department of Pharmaceutical Chemistry, University of Debrecen, Egyetem tér 1, H-4032 Debrecen, Hungary; 4 Department of Inorganic and Analytical Chemistry, University of Debrecen, Egyetem tér 1, H-4032 Debrecen, Hungary; 5 Department of Medical Chemistry, Faculty of Medicine, University of Debrecen, Egyetem tér 1, H-4032 Debrecen, Hungary; 6 Department of Botany, University of Debrecen, Egyetem tér 1, H-4032 Debrecen, Hungary

**Keywords:** *Pseudomonas aeruginosa*, lectin, fucosylated lactose, multivalency, saturation transfer difference NMR

## Abstract

The Gram-negative bacterium *Pseudomonas aeruginosa* is an important opportunistic human pathogen associated with cystic fibrosis. *P. aeruginosa* produces two soluble lectins, the d-galactose-specific lectin PA-IL (LecA) and the l-fucose-specific lectin PA-IIL (LecB), among other virulence factors. These lectins play an important role in the adhesion to host cells and biofilm formation. Moreover, PA-IL is cytotoxic to respiratory cells in the primary culture. Therefore, these lectins are promising therapeutic targets. Specifically, carbohydrate-based compounds could inhibit their activity. In the present work, a 3-*O*-fucosyl lactose-containing tetravalent glycocluster was synthesized and utilized as a mutual ligand of galactophilic and fucophilic lectins. Pentaerythritol equipped with azido ethylene glycol-linkers was chosen as a multivalent scaffold and the glycocluster was constructed by coupling the scaffold with propargyl 3-*O*-fucosyl lactoside using an azide-alkyne 1,3-dipolar cycloaddition reaction. The interactions between the glycocluster and PA-IL or PA-IIL were investigated by isothermal titration microcalorimetry and saturation transfer difference NMR spectroscopy. These results may assist in the development of efficient anti-adhesion therapy for the treatment of a *P. aeruginosa* infection.

## 1. Introduction

Lectins are specific carbohydrate-binding proteins of non-immune origins. A common role of lectins of pathogens is an involvement in the recognition and adhesion between pathogens and hosts which are crucial processes in the development of infections [1]. Therefore, lectins from pathogens may be virulence factors and thus interesting therapeutic targets [2]. Lectins are often multivalent proteins, forming oligomers and/or containing several binding sites for interaction with other molecules. They frequently display an avidity effect resulting in a significantly increased affinity towards their native ligands, glycosylated surfaces. Thus, multivalent carbohydrate-based compounds can be suitable inhibitors of their effect [3].

Lectins are considered to be involved in the pathogenesis of the chronic pulmonary infections associated with cystic fibrosis (CF). Infection and colonization of the lungs by opportunistic pathogens is essentially the main cause of mortality among individuals suffering from this hereditary disease [4]. The most widespread pathogen associated with CF is *Pseudomonas aeruginosa* [5]. Multidrug-resistant *P. aeruginosa* (PA) is included in the WHO list of pathogens as a critical priority bacterium that poses a particular threat in hospitals and causes serious and often fatal infections, such as bloodstream infections and pneumonia. Therefore, there is an urgent need to discover novel antimicrobial agents and develop therapeutic methods based on anti-adhesion targeting the lectins of PA bacteria. This bacterium produces, among other virulence factors, two soluble lectins: PA-IL (LecA) [6,7] and PA-IIL (LecB) [8]. Both proteins are tetrameric, calcium-dependent and have one carbohydrate binding site per monomer, however they differ in sequence, structure and binding specificity. PA-IL is a d-galactose-specific lectin containing one calcium ion in the binding site [9]. PA-IIL is an l-fucose-specific protein with two calcium ions in the binding site [10]. This lectin is also involved in adhesion and biofilm formation as well as its ability to block ciliary beating of epithelial cells was observed [11]. The supposed functions of PA-IL include adhesion to host cells, biofilm formation, cytotoxicity and cellular invasion [11,12,13,14].

Various types of multivalent galactose-containing inhibitors were synthesized and tested against PA-IL, including glycoconjugates with different scaffolds, modified nanoparticles and micelles [15,16,17,18,19,20,21,22,23,24]. Diverse carbohydrate-based inhibitors have also been designed against PA-IIL [21,24,25,26,27,28,29,30]. Based on our state of knowledge, the effective inhibition of *P. aeruginosa* infection in vivo requires the development of novel glycomimetic for anti-adhesion therapy binding simultaneously to lectins PA-IL and PA-IIL. This could be achieved by the administration of two types of inhibitors optimized either for PA-IL or PA-IIL. A promising alternative approach is the utilization of heteroglycoclusters, i.e., compounds containing diverse carbohydrates, which could interact with both lectins. Glycosylated peptide [31] and cyclopeptide [32] dendrimers carrying mixed terminal carbohydrates and gold nanorods decorated with two types of glycomimetic polymers [33] were proposed and examined as dual inhibitors of *P. aeruginosa* lectins. Moreover as mutual ligands, [2] rotaxane heteroglycoclusters combining galactose and fucose subunits [34], mannosyl-centered homo- and heteroglycocluster bearing four α-l-fucoses and four β-d-galactoses and their oligonucleotide conjugates [35], homo- and hetero-bifunctional glycodendrimers ending with up to 16 fucoside and/or galactoside residues have also been prepared [36]. Another important issue of this area is related to the effect of multivalency. It is known from the literature that PA-IL is sensitive to multivalent effect [26], but in contrast, binding studies with glycofullerenes containing up to 24 fucose residue proved that lectin PA-IIL is not sensitive to cluster effect [27].

**Scheme 1:**
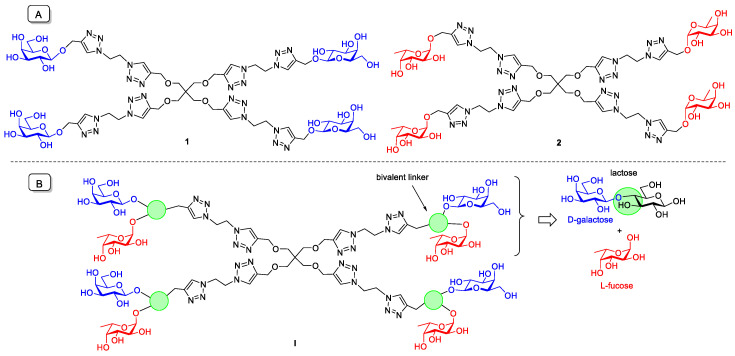
(**A**) Previous results: specific ligands **1** and **2** with selectivity to either galactose-binding lectin PA-IL or fucose-binding lectin PA-IIL. (**B**) This work: the designed mutual ligand of PA-IL and PA-IIL.

Over decades, synthesis of potential ligands of lectins from bacterial or fungal origin has been the main priority of our research. In previous studies, we demonstrated that tetravalent d-galactoside **1 [37]** (Scheme 1) and its *S*- or *Se*-glycoside analogues [38] inhibited galactophilic PA-IL and we proved that compound **1** decreased *P. aeruginosa* adhesion to bronchial cells ex vivo. We also found that l-fucoside **2** [39,40] was a potent inhibitor of fucophilic lectin PA-IIL and has anti-adhesion properties on cellular level. The most promising anti-adhesion properties and efficacy were achieved when compound **1** was applied in combination with a tetravalent fucoside [37]. This mixture was also able to decrease adhesion of *P. aeruginosa* cells to bronchial human cells in the ex vivo adhesion assay.

In order to obtain an even more effective inhibitor of *P. aeruginosa*, we decided to synthesize a dual ligand using the same tetravalent architecture, which contains both d-galactose and l-fucose units (Scheme 1). However, unlike heteroglycoclusters, which alternately contain either fucose or galactose units on a multivalent core, we envisioned the synthesis of a combined glycocluster that contains both galactose and fucose units at each end of the multivalent scaffold. This structure can easily be synthesized if L-fucose is coupled to the natural disaccharide lactose and the trisaccharide thus obtained is attached to the multivalent scaffold (Scheme 1B). The added value of this approach is the easy and efficient synthesis of a mutual ligand of both lectins of *P. aeruginosa*. A tetravalent oligoglycocluster containing four d-galactoside and four l-fucoside units, such as **I** (Scheme 1B), may provide an excellent opportunity to test the viability of the above idea. In order to characterize the potencies of the newly synthesized glycocluster and to disclose the potential of cluster effect, if any, in the carbohydrate–protein interaction, we followed the strategy used in our previous studies, where the interaction of compounds **1** and **2** with galactophilic [37] and fucophilic lectin [39,40] were investigated by isothermal titration microcalorimetry (ITC) and saturation transfer difference NMR spectroscopy (STD-NMR) methods.

## 2. Results

In the present paper, the efficient synthesis of a 3-*O*-fucosyl lactose-presenting tetravalent glycocluster is described and testing as an inhibitor of lectins PA-IL and PA-IIL is probed. The interactions of this molecule with both lectins were quantified by isothermal titration microcalorimetry and also characterized by saturation transfer difference NMR spectroscopy.

### 2.1. Synthesis of Tetravalent Fucosylated Lactoside

**Scheme 2:**
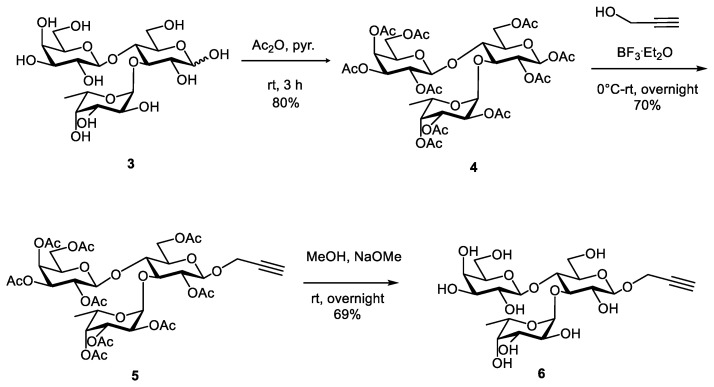
Synthesis of the propargyl glycoside of 3-*O*-α-l-fucosyl lactose.

In order to prepare an appropriate building block to the glycocluster, 3-*O*-α-l-fucosyl lactose (**3**) was synthesized according to the literature [41] and then peracetylated to obtain **4**. Trisaccharide **4** was converted into propargyl glycoside using propargyl alcohol and boron trifluoride diethyl etherate as promoter to result in compound **5** with a yield of 70% (Scheme 2.). The acetyl esters were removed by Zemplén deacetylation to provide the unprotected propargyl glycoside **6**, ready for azide-alkyne click reaction.

**Scheme 3:**
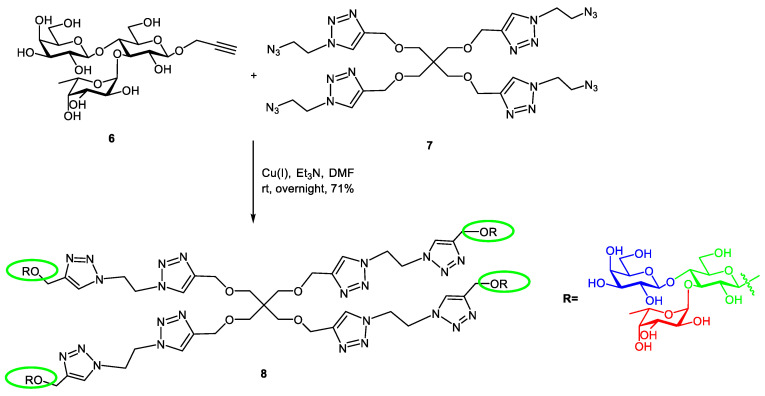
Synthesis of the tetravalent glycocluster **8** having 3-*O*-fucosyl lactoside units.

Similarly to our recent works [37,38,39,40], a pentaerythritol core **7** [40] equipped with azido ethylene glycol linkers was chosen as a multivalent scaffold. The oligoglycocluster was constructed by copper(I)-mediated 1,3-dipolar azide-alkyne cycloaddition (CuAAC) click reaction. Coupling of the tetravalent scaffold **7** with trisaccharide **6** resulted in the final glycocluster **8** with excellent yield (Scheme 3). Compounds **6** and **8** were tested as potential mutual ligands of the galactophilic PA-IL and the fucophilic PA-IIL lectins.

### 2.2. Thermodynamics of the Interactions

**Figure 1:**
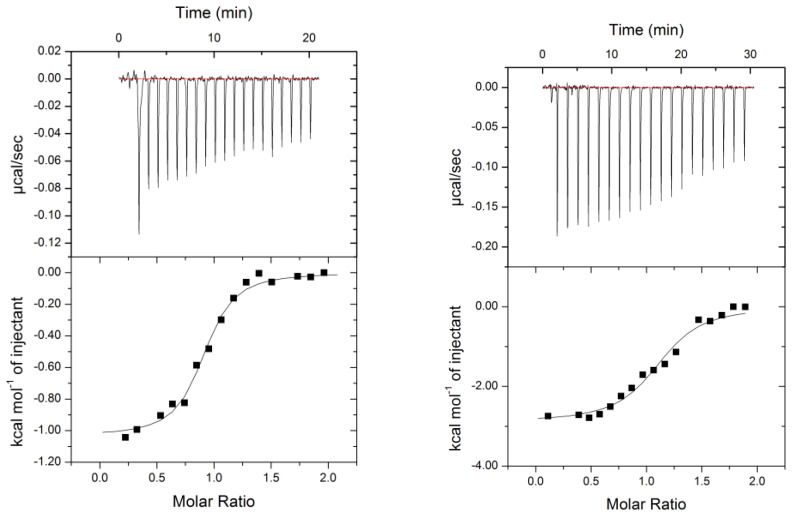
Interaction of compound **8** with PA-IL (**left**) and PA-IIL (**right**).

The results of isothermal titration calorimetry measurements providing thermodynamic characterization of the interaction of compound **8** with PA-IL and PA-IIL lectins (Figure 1) and compound **6** with PA-IL are summarized in Table 1 and Table 2, respectively. The corresponding data of the relevant monomeric ligands (D-Gal, Me ß-D-Gal, L-Fuc and Me ß-L-Fuc), together with those of compounds **1** and **2** are given for comparison. Compound **6** showed only slightly better affinity to PA-IL than the monomers (D-Gal or Me ß-D-Gal). On the contrary, the tetravalent glycocluster **8** was able to bind to PA-IL with two orders of magnitude higher affinity than the relevant monomers (Table 1), and its potency is commensurate with tetragalactocluster **1**. These data unequivocally confirm the presence of a multivalent effect in the binding of the tetravalent compound **8** to PA-IL. The glycocluster **8** was able to bind to PA-IIL as well with affinity similar to tetrafucocluster **2**, monovalent compound **6** and Me α-L-Fuc, but showed significantly higher affinity than L-fucose. It should be also noted that the K_a_ value of monovalent propargyl glycoside **6** is significantly higher than those of the corresponding natural ligands in case of both lectins due to interaction of the aglycon propargyl group with the lectin binding sites. This interaction was already observed in previous studies between PA-IL and propargyl *Se*-ß-d-galactopyranoside [38] and PA-IIL and propargyl α-l-fucopyranoside [39], respectively. The stoichiometries (*n* value) of interaction in the case of tetravalent compound **8** was close to 1 in both cases (Table 1 and Table 2, Figure 1), suggesting that one molecule of inhibitor is recognized by all binding sites of tetravalent PA-IL and PA-IIL. No aggregation was observed during or after calorimetry measurement implying that the binding is either intramolecular (within the same PA tetramer) or the particles resulting from intermolecular binding are too small to form visually observable aggregates in the concentration range of the measurements.

### 2.3. STD-NMR Studies: Binding of Compounds ***6*** and ***8*** to PA-IL and PA-IIL Lectins Characterized by ^1^H STD Competition NMR Experiments

**Figure 2:**
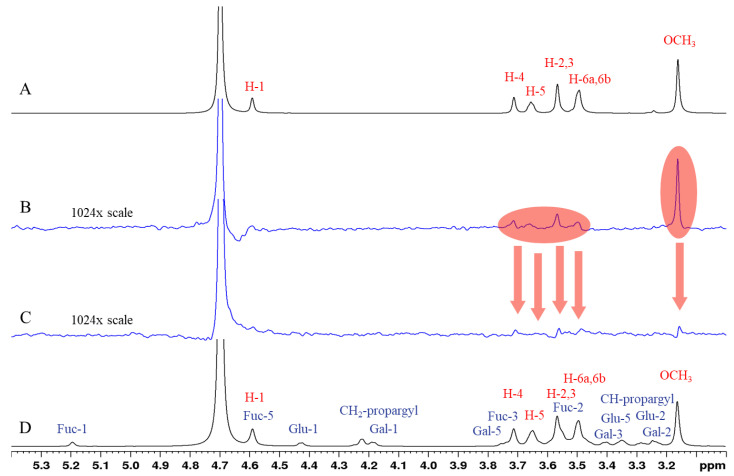
(**A**) ^1^H and (**B**) STD NMR spectra of 4 mM Meα-D-Gal in the presence of 40 μM PA-IL monomer in D_2_O (700 MHz, 20 mM Tris-d_11_, 150 mM NaCl, 5 mM CaCl_2_, pH = 7.5, T = 283 K). (**C**) STD and (**D**) ^1^H spectra of the same sample upon addition of 4 mM propargyl glycoside of 3-fucosyl lactose (compound **6**). The proton signals of the reference ligand are assigned with red color, while those of the competitor are indicated in blue. The well-separated *O*-methyl signal of Me α-D-Gal serves as excellent indicator for monitoring the competition between the reference ligand and compound **6**. The fractional reduction of the methyl STD intensities (from 100% to 12%) measured in 1:1 molar mixture of the competitor and reference ligand indicates the higher affinity of compound **6**.

Saturation transfer difference NMR spectroscopy [44,45,46] is one of the most sensitive and versatile ligand-observed methods for detecting binding by NMR in the low-affinity (*K*_D_ = μM–mM) range. The utility and applicability of STD method can be further extended if it is combined with competition binding experiments. Particularly, competition STD NMR experiments allows the detection of the binding over a wide affinity range, including both low- and high-affinity ligands, provides quick qualitative information on relative binding affinities and allows to characterize the binding interaction as being of specific or non-specific nature. The only requirement for the competition STD NMR experiment to work is the availability of a known low-affinity reference ligand of the protein, which has at least one STD signal well-separated from the STD resonances of other potential ligands in the mixture. In the present study, in order to characterize the binding and also the binding specificity of the monovalent fucosylated lactoside (compound **6**) and the tetravalent fucosylated lactoside (compound **8**) with PA-IL and PA-IIL, we performed competition STD NMR experiments using Meα-D-Gal and Me α-D-Man as STD reference ligands with known low-affinity to both lectins. Figure 2A,B show the 1D ^1^H and STD NMR spectra of the reference ligand (4 mM) in the presence of PA-IL lectin (40 μM), respectively. The STD signals (highlighted by red ellipsoids) confirm the binding of Meα-D-Gal and indicate the ring protons’ and also the methyl group’s involvement in the interaction. Upon addition of compound **6**, the STD signals of the reference ligand are significantly reduced (Figure 2C). This result clearly demonstrates that compound **6** occupies the same or at least part of the same binding site on PA-IL as the natural reference ligand. Moreover, considering the 1:1 molar ratio of the reference and competing ligands in the sample, the substantial intensity drop (from 100% to 12%) measured on the methyl signal suggests that compound **6** shows significantly higher affinity towards PA-IL than the reference Me α-D-Gal used in the competition assay.

**Figure 3:**
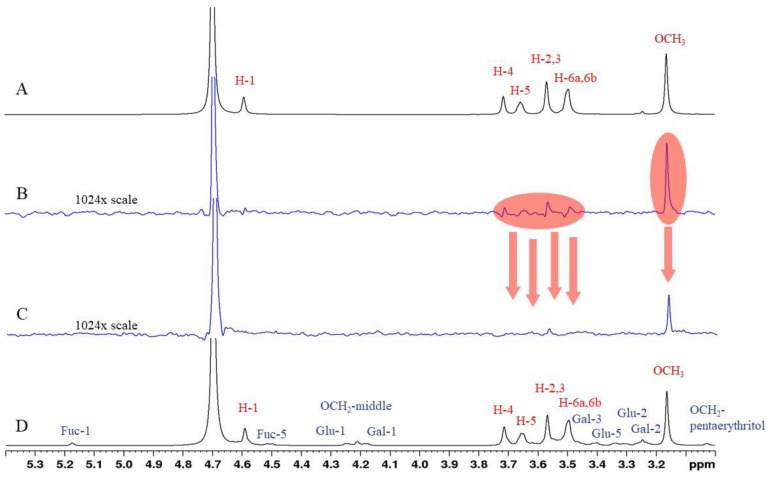
(**A**) ^1^H and (**B**) STD NMR spectra of 4 mM Meα-D-Gal in the presence of 40 μM PA-IL monomer in D_2_O (700 MHz, 20 mM Tris-d_11_, 150 mM NaCl, 5 mM CaCl_2_, pH = 7.5, T = 283 K). (**C**) STD and (**D**) ^1^H spectra of the same sample titrated with 0.4 mM tetravalent 3-fucosyl lactose derivative (compound **8**). The proton signals of the reference ligand are assigned with red color, while those of the competitor are indicated in blue. The fractional reduction of the methyl STD intensities (from 100% to 56%) measured in 1:10 molar mixture of the competitor and reference ligand confirms the binding and higher affinity of compound **8**.

Interestingly, in the case of the tetravalent compound **8**, we discovered a strong aggregation tendency for both PA-IL and PA-IIL lectins, approximately after 12 h, even at a relatively low concentration of the ligand (0.8 mM). The aggregation can most likely be explained by cross-linking induced via the tetravalent ligand. Therefore, in order to avoid the undesired aggregation (precipitation) of PA-IL lectin, compound **8** was introduced at significantly lower concentration into the mixture (reference ligand (4 mM), compound **8** (0.4 mM) and lectin (40 μM), respectively, i.e., in 100:10:1 molar ratio). Figure 3C illustrates the drop (or disappearance) of STD signals of the reference Meα-D-Gal ligand upon addition of compound **8**. The fractional reduction (from 100% to 56%) measured on the methyl peak in 1:10 molar mixture of the competitor and reference ligand confirms the binding and higher affinity of compound **8**.

**Figure 4:**
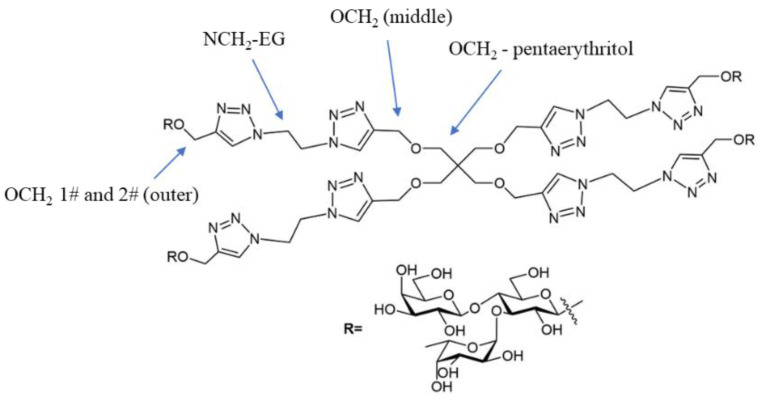
Structure of compound **8** and explanation of relevant structural moieties.

Similar competition STD NMR experiments were performed with compounds **6** and **8** in the presence of the other lectin, PA-IIL using Meα-D-Man as the reference ligand. It is known from the literature [47] that this molecule also binds to the fucosyl recognition domain of the lectin but with lower affinity than Me α-L-Fuc. As before, the reduction in STD signal of the reference ligand confirmed the competitive binding of both the mono- and tetravalent ligands (representative spectra are shown in Figures S1 and S2 in the Supporting Information). In addition, specific evidence of lectin precipitation due to cross-linking induced by the tetravalent ligand (compound **8**) is given in Table S1 of SI, where the relative STD signal intensities measured on the methyl signal of the reference ligand are given as a function of the concentration of compound **8** (Figure 4). Moreover, STD experiments under identical conditions were repeated on the last sample, containing compound **8** in 4 mM concentration and STD signal intensities were monitored as a function of time. As expected, the gradual reduction of the methyl STD intensity of the reference ligand upon adding compound **8** up to 0.4 mM confirmed the competitive binding of the tetravalent ligand. However, adding more ligand to the mixture, the reference methyl STD signal started to increase, which can be most likely due to the aggregation and subsequent precipitation of the lectin, leading to higher reference ligand: lectin molar ratio, whilst the corresponding ratio for the tetravalent ligand (compound **8**) is shifted in the opposite direction due to its involvement in cross-linking.

## 3. Discussion

As a result, click-chemistry approach was applied to synthesize tetravalent glycodendrimers bearing four 3-*O*-α-l-fucosylated lactose units, as potential ligand of lectins PA-IL (LecA) and PA-IIL (LecB) isolated from *P. aeruginosa,* with fucophilic and galactophilic character, respectively. To the best of our knowledge, no example can be found in the literature where oligosaccharides are coupled to oligovalent structure decorated with terminal d-galactose and l-fucose units. The ability of the synthetized tetravalent glycoclusters to interact with lectins PA-IL (LecA) and PA-IIL (LecB) from *Pseudomonas aeruginosa* was proved, and the tetravalent compound **8** was found to be suitable ligand of the lectins *in vitro*, with significantly better binding affinity than simple d-galactose or l-fucose. Although affinity of the novel compound is commensurate with previously synthesized tetravalent galacto- and fucocluster (**1** and **2**), we expect that a combined synergic effect might evolve ex vivo or cellular level. The ITC measurements established that the tetravalent glycocluster **8** bound to lectin PA-IL with *K*_a_ value of two orders of magnitude higher than their natural ligands, which confirms the presence of cluster effect for compound **8**. On the contrary, the compound **8** binding affinity toward lectin PA-IIL was similar to that of Me α-L-Fuc, propargyl 3-*O*-fucosyl lactose **6** and only slightly more than 20-times better than that of l-fucose. These results clearly confirm the hypothesis that PA-IL is sensitive to a multivalent effect, while PA-IIL seems to be independent from the cluster effect. Moreover, the stochiometric data shown (*n* values are around 1 in both cases) implicate, that there are interactions between the ligand and the protein in a one-to-one complex, meaning all binding sites of lectin are saturated by the ligand at the low-concentration range. On the other hand, bindings involving more lectins leading to cross-linking and self-aggregation could be expected under therapeutic conditions, at higher concentration range. In such case the mutual tetravalent glycocluster **8** exposing either the galactose or the fucose unit may allow a simultaneous binding to any of the two lectins on the bacterial surface. Using competition STD-NMR experiments, we have unambiguously confirmed the specific binding of the mono- and tetravalent ligands to both lectins. The reduction (or disappearance) in the STD signals of the low-affinity reference ligand, Meα-D-Gal or Meα-D-Man upon titration proved that both ligands compete with the reference ligand for the same (or at least overlapping) binding site of PA-IL and PA-IIL lectins. In addition, the relative intensity reduction in STD signals of the reference ligand allowed a qualitative rank ordering of the ligands and confirmed, in agreement with the ITC results, the higher affinity of the tetravalent ligand. The unusual concentration and time dependence of STD effects observed on the reference ligand signals upon titrating with compound **8** in the presence of either PA-IL or PA-IIL suggests the occurrence of cross-linking of lectins through the tetravalent ligand leading to the formation of larger aggregates and subsequent partial precipitation of lectins. The anti-adhesion therapy requires extremely high concentrations (up to 0.1 M) of ligand [48], when self-aggregation and cross-linking of lectins caused by a multivalent ligand is probably the most preferable interaction. Application of a mutual multivalent ligand may promote to evolve such intermolecular interactions via the terminal galactose and fucose residues furnished by different arms of the tetravalent architecture, making potentially feasible the simultaneous binding of both lectins on the bacterial surface to prevent colonization. However, further studies at cellular levels are still needed to assess the potential applications of these glycomimetics with great anti-adhesive properties in animal studies [49] or human therapy of PA infections [48].

## 4. Materials and Methods

### 4.1. General Methods

Optical rotations were measured at room temperature with a Perkin-Elmer 241 automatic polarimeter (PerkinElmer, Waltham, MA, USA). TLC analysis was performed on Kieselgel 60 F_254_ (Merck, Kenilworth, NJ, USA) silica gel plates with visualization by immersing in a sulfuric acid solution (5% in EtOH, VWR International Ltd., Radnor, PA, USA) followed by heating. Column chromatography was performed on silica gel 60 (Merck 0.063–0.200 mm), flash column chromatography was performed on silica gel 60 (Merck 0.040–0.063 mm). Gel filtration was performed on Sephadex G-25, using methanol as the eluent. Organic solutions were dried over MgSO_4_ and concentrated under vacuum. The ^1^H (400 and 500 MHz) and ^13^C NMR (100.28, 125.76 MHz) spectra were recorded with Bruker DRX-400 and Bruker Avance II 500 spectrometers (Bruker, Billerica, MA, USA). Chemical shifts are referenced to Me_4_Si or DSS (0.00 ppm for ^1^H) and to solvent signals (CDCl_3_: 77.00 ppm, CD_3_OD: 49.15 ppm for ^13^C). MS (MALDI-TOF) analysis was carried out in positive reflectron mode with a BIFLEX III mass spectrometer (Bruker, Billerica, MA, USA) with delayed-ion extraction. The matrix solution was a saturated solution of 2,4,6-trihydroxy-acetophenone (THAP) in CH_3_CN. ESI-QTOF MS measurements were carried out on a maXis II UHR ESI-QTOF MS instrument (Bruker, Billerica, MA, USA), in positive ionization mode. The following parameters were applied for the electrospray ion source: capillary voltage: 3.6 kV; end plate offset: 500 V; nebulizer pressure: 0.5 bar; dry gas temperature: 200 °C and dry gas flow rate: 4.0 L/min. Constant background correction was applied for each spectrum, the background was recorded before each sample by injecting the blank sample matrix (solvent). Na-formate calibrant was injected after each sample, which enabled internal calibration during data evaluation. Mass spectra were recorded by otofControl version 4.1 (build: 3.5, Bruker, Billerica, MA, USA) and processed by Compass DataAnalysis version 4.4 (build: 200.55.2969) (Bruker, Billerica, MA, USA). Lectins in recombinant form were produced and purified as previously described [9, 10] by Wimmerová group (Brno, Masaryk University).

#### 4.1.1. Compound **4**

Compound **3** (1.000 g, 2.00 mmol) was dissolved in pyridine (5 mL) and Ac_2_O (5 mL) was added drop-wise under ice-bath cooling then after one hour it was heated to 60 °C for three hours. The crude product was poured into ice, extracted with CH_2_Cl_2_ (2 × 200 mL), washed with water (50 mL), satd. aq. NaHCO_3_ (50 mL) and brine (50 mL), then it was dried over MgSO_4_, filtered and evaporated, then purified by flash column chromatography (95:5 CH_2_Cl_2_:MeOH) to give compound **4** (1.49 g, 80%) as a colourless syrup. R*_f_* 0.33 (92:8 CH_2_Cl_2_: acetone). The crude product was used for the next step without characterization.

#### 4.1.2. Compound **5**

To a stirred solution of compound **4** (1.000 g, 1.10 mmol) dissolved in *N*,*N*-dimethylformamide (50 mL) propargyl alcohol (95 μL, 1.5 equiv.) was added. The reaction mixture was cooled to 0 °C under argon atmosphere and BF_3_^.^Et_2_O (204 μL, 1.5 equiv.) was added and stirred overnight at room temperature. The reaction mixture was diluted with CH_2_Cl_2_ (200 mL), washed with water (50 mL), satd. aq. NaHCO_3_ (50 mL) and brine (50 mL), then it was dried over MgSO_4_, filtered and evaporated. The crude product was purified by flash column chromatography (*n*-hexane:acetone 6:4) to give compound **5** (700 mg, 70%) as a colourless syrup. [α]^24^_D_— −90.0 (*c* 0.21, CHCl_3_); R*_f_* 0.38 (9:1 CH_2_Cl_2_: acetone); MALDI-TOF (positive ion): *m*/*z* calcd for C_39_H_52_NaO_24_: 931.2695 [M + Na]^+^ Found: 931.2690. ^1^H NMR (400 MHz, CDCl_3_) δ 5.40 (ddd, *J* = 6.3, 3.4, 1.1 Hz, 2H), 5.35 (d, *J* = 4.0 Hz, 1H), 5.19 (dd, *J* = 10.9, 3.4 Hz, 1H), 5.12–4.90 (m, 5H), 4.64 (dd, *J* = 12.2, 2.1 Hz, 1H), 4.58 (d, *J* = 8.0 Hz, 1H), 4.54 (dd, *J* = 11.5, 6.5 Hz, 1H), 4.48 (d, *J* = 8.0 Hz, 1H), 4.34–4.25 (m, 3H), 4.08 (dd, *J* = 12.2, 4.2 Hz, 1H), 3.97 (t, *J* = 9.4 Hz, 1H), 3.92–3.84 (m, 2H), 3.54–3.45 (m, 1H), 2.19 (s, 3H), 2.15 (s, 3H), 2.15 (s, 3H), 2.08 (s, 3H), 2.07 (s, 3H), 2.05 (s, 3H), 2.03 (s, 3H), 1.96 (s, 3H), 1.98 (s, 3H), 1.23 (d, *J* = 6.5 Hz, 3H). ^13^C NMR (101 MHz, CDCl_3_) δ 171.1, 170.7, 170.5, 170.4, 170.2, 169.9, 169.7, 169.6, 168.9, 4× CO), 100.5, 98.1, 95.4 (C-1, C-1′, C-1″), 78.2 (C_q_, propargyl), 75.2, 74.0, 73.2, 71.3, 70.9, 70.9, 68.9, 68.0, 67.8, 66.6, 64.1 (Cskeleton, CH_2_O-), 61.2, 60.6 (C-6, C-6′), 55.7 (-OCH_2_), 20.9, 20.8, 20.7, 20.7, 20.6, 20.5, 20.4 (CH_3_, acetyl), 15.7 (Fuc-CH_3_).

#### 4.1.3. Compound **6**

Compound **5** (700 mg, 0.77 mmol) was dissolved in MeOH (10 mL), catalytic amount of NaOMe (pH~9) was added and stirred overnight at room temperature. The reaction mixture was neutralized with Amberlite IR-120 H^+^ ion-exchange resin, filtered and evaporated, then the crude product was purified by flash column chromatography (83:17 CH_3_CN:H_2_O) to give compound **6** (279 mg, 69%) as a colourless syrup. [α]^24^_D_ −67.2 (*c* 0.22, MeOH); R*_f_* 0.58 (7:3 CH_3_CN:H_2_O); ^1^H-NMR (700 MHz, D_2_O, 298 K): *δ* = 5.45 (d, *J* = 3.9 Hz, 1H, H_Fuc_-1), 4.83 (q, 1H, H_Fuc_-5); 4.67 (d, *J* = 8.0 Hz, 1H, H_Glu_-1), 4.51-4.42 (overlapping signals, 3H, H_Gal_-1, CH_2_-propargyl); 4.01 (dd, 1H, H_Gal_-5), 3.97 (dd, 1H, H_Fuc_-3), 3.93–3.86 (overlapping signals, 2H, H_Gal_-4, H_Glu_-4), 3.86–3.70 (overlapping signals, 7H, H_Fuc_-4, H_Gal_-6ab, H_Glu_-6ab, H_Glu_-3, H_Fuc_-2) 3.66 (dd, 1H, H_Gal_-3), 3.63–3.57 (overlapping signals, 2H, H_Glu-5_, CH_-_propargyl), 3.55 (t, 1H, H_Glu_-2), 3.49 (t, 1H, H_Gal_-2), 1.19 (d, *J* = 6.6 Hz, 3H, Fuc-CH_3_) ppm. ^13^C-NMR (175 MHz, D_2_O, 298 K): δ = 101.9 (C_Gal_-1), 100.6 (C_Glu_-1), 98.5 (C_Fuc_-1), 77.1 (C_Glu_-3), 75.2 (CH-propargyl), 74.3 (C_Glu_-2), 72.6 (C_Glu_-4), 72.5 (C_Gal_-3), 72.0 (C_Fuc_-4), 71.2 (C_Gal_-2), 69.3 (C_Fuc_-3), 68.4 (C_Gal_-4), 68.1 (C_Fuc_-2), 66.6 (C_Fuc_-5), 61.5 (C_Glu_-5), 61.5 (C_Glu_-6), 59.8 (C_Gal_-5), 59.7 (C_Gal_-6), 57.0 (CH_2_-propargyl), 15.4 (Fuc-CH_3_) ppm. MALDI-TOF (positive ion): *m*/*z* calcd for C_21_H_34_NaO_15_: 549.1795 [M + Na]^+^ Found: 549.1798.

#### 4.1.4. Compound **8**

Et_3_N (12 μL, 1 equiv./alkyne) and Cu(I)I (1.6 mg, 0.1 equiv./alkyne) were added to a stirred solution of azide compound **6** (59 mg, 0.08 mmol) and alkyne **5** (212 mg, 0.403 mmol) in MeOH (5 mL) under an argon atmosphere and stirred overnight at room temperature. The reaction mixture was evaporated, and the crude product was purified by flash column chromatography (6:4 CH_3_CN:H_2_O) to give compound **8** (159 mg, 71%) as a colourless syrup. [α]^24^_D_ −72.8 (*c* 0.19, H_2_O); R*_f_* 0.26 (6:4 CH_3_CN:H_2_O). ^1^H-NMR (700 MHz, D_2_O, 298 K) (based on Figure 4) *δ* = 7.89, 7.82 (2× s, 8H, 8× CH triazole), 5.43 (d, *J* = 4.1 Hz, 4H, H_Fuc_-1), 4.98–4.93 (overlapping signals, 16H, 8× NCH_2 -_EG), 4.88 (d, 4H, OCH_2_ #1 outer); 4.81 (q, 4H, H_Fuc_-5); 4.51–4.41 (overlapping signals, 16H, 4× H_Gal_-1, 4× H_Glu_-1, 4× OCH_2_ middle); 4.00–3.94 (overlapping signals, 8H, 4× H_Gal_-5, 4× H_Fuc_-3); 3.92 (d, 4H, H_Gal_-4), 3.89–3.70 (overlapping signals, 32H, 4× H_Glu_-4, 4× H_Gal_-6ab, 4× H_Glu_-6ab, 4× H_Glu_-3, 4× H_Fuc_-4, 4× H_Fuc_-2) 3.66 (dd, 4H, 4× H_Gal_-3), 3.60 (m, 4H, 4× H_Glu_-5), 3.53-3.47 (overlapping signals, 8H, 4× H_Glu_-2, 4× H_Gal_-2), 3.29 (s, 8H 4× OCH_2_-pentaerythritol), 1.18 (d, *J* = 6.5 Hz, 12H, 4× CH_3_) ppm. ^13^C-NMR (175 MHz, D_2_O, 298 K): δ = 126.2 and 125.5 (8C, 8× CH triazole), 101.8 (4C, 4× C_Gal_-1), 101.3 (4C, 4× C_Glu_-1), 98.3 (4C, 4× C_Fuc_-1), 77.0 (4C, 4× C_Glu_-3), 75.4 (4C, 4× C_Glu_-6), 74.4 (4C, 4× C_Glu_-2), 72.7 (4C, 4× C_Glu_-4), 72.5 (4C, 4× C_Gal_-3), 72.0 (4C, 4× C_Fuc_-4), 71.2 (4C, 4× C_Gal_-2), 69.2 (4C, 4× C_Fuc_-3), 68.5 (1C, 1× C_q_, pentaerythritol), 68.4 (4C, 4× C_Gal_-4), 68.1 (4C, 4× C_Fuc_-2), 68.1 (4C, 4× OCH_2_-pentaerythritol), 66.5 (4C, 4× C_Fuc_-5), 63.1 (4C, 4× OCH_2_ middle), 61.4 (4C, 4× C_Glu_-5), 59.8 (4C, 4× C_Gal_-5) and (4C, 4× C_Gal_-6), 50.0 and 49.8 (8C, 8× NCH_2_-EG), 44.5 (4C, 4× “C” inner triazole), 43.9 (4C, 4× “C” outer triazole), 15.4 (4C, 4× CH_3_) ppm. ESI-HRMS: *m*/*z* calcd for C_109_H_172_N_24_Na_2_O_64_: 2864.0840 [M + 2Na]^2+^ Found: 1444.0378.

### 4.2. Isothermal Microcalorimetry

All experiments were performed on MicroCal iTC200 (Malvern Instruments, Malvern, UK) at 25 °C. The freeze-dried lectins were dissolved in 0.1 M Tris/HCl, 500 μM CaCl_2_, pH 7.5, and equilibrated at room temperature for 1 h before ITC measurements. Mono- and multivalent compounds **6** and **8** were diluted in the same buffer and used in 0.1 mM concentrations. Aliquots of 2 µL of the compounds were added automatically to the 0.01 mM PA-IL or 0.011 mM PA-IIL in the calorimeter cell. Blank experiments (injections of compounds to the buffer) were performed and heat responses were subtracted. Integrated heat effects were analyzed by global fitting of the data obtained from three independent titrations for each tested compound by non-linear regression using a single site-binding model (Microcal Origin 7, Malvern Instruments, Malvern, UK). The fitted data yielded the association constant (K_a_), the enthalpy of binding (ΔH) and entropy of binding (ΔS). Other thermodynamic parameters, i.e., changes in free energy (ΔG), were calculated from the equation: ΔG = ΔH−TΔS = −RTln K_a_, where T is the absolute temperature and R = 8.314 J mol^−1^ K^−1^.

### 4.3. ^1^H STD Competition NMR Experiments

All STD NMR experiments were performed on a Bruker Avance Neo 700 MHz spectrometer (Bruker, Billerica, MA, USA) equipped with 5 mm z-gradient TCI inverse detection Prodigy probe. The pseudo 2D pulse program stddiff·3 available in the pulse sequence library of TopSpin 4.0.5 was used for the measuments. Samples were prepared in D_2_O/20 mM deuterated Tris-buffer (150 mM NaCl, 5 mM CaCl_2_, pH = 7,5, T = 283 K). The protein resonances were selectively irradiated using Gauss (90°) pulses with a length of 50 ms. The total duration of saturation was set to 3 s. The on- and off-resonance saturation frequency of the selective pulse was switched between −0.5 and −40 ppm after each scan and the corresponding FIDs were collected in separate memories for subsequent processing and generation of STD spectra. A spin-lock pulse with 10 kHz field and 50 ms duration was used to suppress the residual protein signals. Reference experiments were carried out to assure the absence of direct irradiation of the ligand. STD spectra were acquired with 3200 transients and 16k data points and zero filled up to 64k data prior processing. To increase the S/N ratio the FIDs were multiplied with an exponential function (lb = 6 Hz). The samples used for STD measurements contained 40 μM lectin (PA-IL or PA-IIL), 4 mM Meα-D-Gal or Meα-D-Man as references, and 4mM compound **6** (1:100:10 molar ratio) or 0.4 mM compound **8** (1:10:100), respectively.

## 5. Conclusions

A highly efficient synthesis of a mutual, 3-*O*-fucosylated lactose-containing tetravalent ligand of both lectins isolated from *Pseudomonas aeruginosa* was developed. The potency of the mutual ligand was compared to the potency of mixed ligands, when tetravalent fucoside and galactoside were used in combination. Although the novel mutual tetravalent glycocluster did not show the expected higher potency *in vitro*, its pertinent binding properties to lectin PA-IL with favorable clustering and cross-linking effects make it a promising candidate for further testing at the cellular level with a potential utilization as a prophylactic agent against bacterial colonization of the lungs.

## Figures and Tables

**Table 1:** Thermodynamic parameters of interactions between lectin PA-IL and ligands.

Inhibitor	Valency	n	K_a_ [10^4^ M^−1^]
d-galactose	1	0.78 ± 0.05	0.68 ± 0.038 [37]
Me β−d-Gal	1		1.4 [42]
Compound **6**	1	0.51 ± 0.08	59 ± 28.4
Compound **1**	4	0.21 ± 0.01	470 ± 54.5 [37]
Compound **8**	4	0.89 ± 0.02	496 ± 104
**Inhibitor**	**Δ** **H [kJ/mol]**	**−T** **Δ** **S [kJ/mol]**	**Δ** **G [kJ/mol]**
d-galactose	−37.8	15.9	−21.9
Me β−d-Gal	−39.0	15.0	−24.0
Compound **6**	−3.48	−29.3	−32.8
Compound **1**	−134	95.7	−38.1
Compound **8**	−4.3	−33.8	−38.1

**Table 2:** Thermodynamic parameters of interactions between lectin PA-IIL and ligands.

Inhibitor	Valency	n	Ka [10^4^ M^−1^]
l-fucose	1	0.96 ± 0.04	12 ± 1 [43]
Me α-L-Fuc	1	0.43± 0.01	235 ± 8.0 [43]
Compound **6**	1	1.12 ± 0.05	344 ± 124
Compound **2**	4	2.1 ± 0.1	310 ± 100 [37]
Compound **8**	4	1.1 ± 0.03	257 ± 66.9
**Inhibitor**	**Δ** **H [kJ/mol]**	**−T** **Δ** **S [kJ/mol]**	**Δ** **G [kJ/mol]**
l-fucose	−27.7	−1.3	−29
Me α-L-Fuc	−41.3	4.9	−36.4
Compound **6**	−36.3	−0.8	−37.1
Compound **2**	−56.9	−25.5	−31.4
Compound **8**	−12.0	−24.3	−36.4

## Data Availability

Not applicable.

## Supplementary Materials

The following are available online at https://www.mdpi.com/article/10.3390/ijms232416194/s1, Figures S1 and S2: ^1^H and STD NMR spectra of ligands **6** and **8** in the presence of PA-IIL. Table S1: Intensity change of the methyl STD signal of the reference compound upon titration with the tetravalent ligand **8**. Figures S3–S10: 1D and 2D NMR spectra of compounds **6** and **8**.
