# Supplementary material for: A Fucosylated Lactose-Presenting Tetravalent Glycocluster Acting as a Mutual Ligand of Pseudomonas aeruginosa Lectins A (PA-IL) and B (PA-IIL)—Synthesis and Interaction Studies
Source: Int J Mol Sci. 2022 Dec 19;23(24):16194. doi: 10.3390/ijms232416194 (PMC9782601; doi:10.3390/ijms232416194)
Supplement: Supplementary file 1 [file ijms-23-16194-s001.zip › ijms-2022668-supplementary.pdf]

International Journal of *Molecular Sciences*

MDPI

SUPPLEMENTARY MATERIAL

A fucosylated lactose-presenting tetravalent glycocluster acting as a mutual ligand of *Pseudomonas aeruginosa* lectin A (PA-IL) and B (PA-IIL)- Synthesis and interaction studies

Magdolna Csávás[1], László Kalmár[2], Petronella Szőke[3],László Bence Farkas[1,4], Bálint Bécsi[5], Zoltán Kónya[5], János Kerékgyártó[6], Anikó Borbás[3], Ferenc Erdődi[5], Katalin E. Kövér[1,4,*]

[1] Research Group for Molecular Recognition and Interaction, Loránd Eötvös Research Network, University of Debrecen, Egyetem tér 1, H-4032 Debrecen, Hungary
[2] Department of Ecology, University of Debrecen, Egyetem tér 1, H-4032 Debrecen, Hungary
[3] Department of Pharmaceutical Chemistry, University of Debrecen, Egyetem tér 1, H-4032, Debrecen, Hungary
[4] Department of Inorganic and Analytical Chemistry, University of Debrecen, Egyetem tér 1, H-4032 Debrecen, Hungary
[5] Department of Medical Chemistry, University of Debrecen, Egyetem tér 1, H-4032 Debrecen, Hungary
[6] Department of Botany, University of Debrecen, Egyetem tér 1, H-4032 Debrecen, Hungary
* Correspondence: kover@science.unideb.hu; +3652-512900

Received: ; Accepted: ; Published:

SUPPLEMENTARY MATERIAL

Figures S1-S2. ^{1}H and STD NMR spectra of ligands **6** and **8** in the presence of PA-IIL.

Table S1. Intensity change of the methyl STD signal of the reference compound upon titration with the tetravalent ligand **8**.

Figures S3-S10. 1D and 2D NMR spectra of compounds 6 and 8.

References

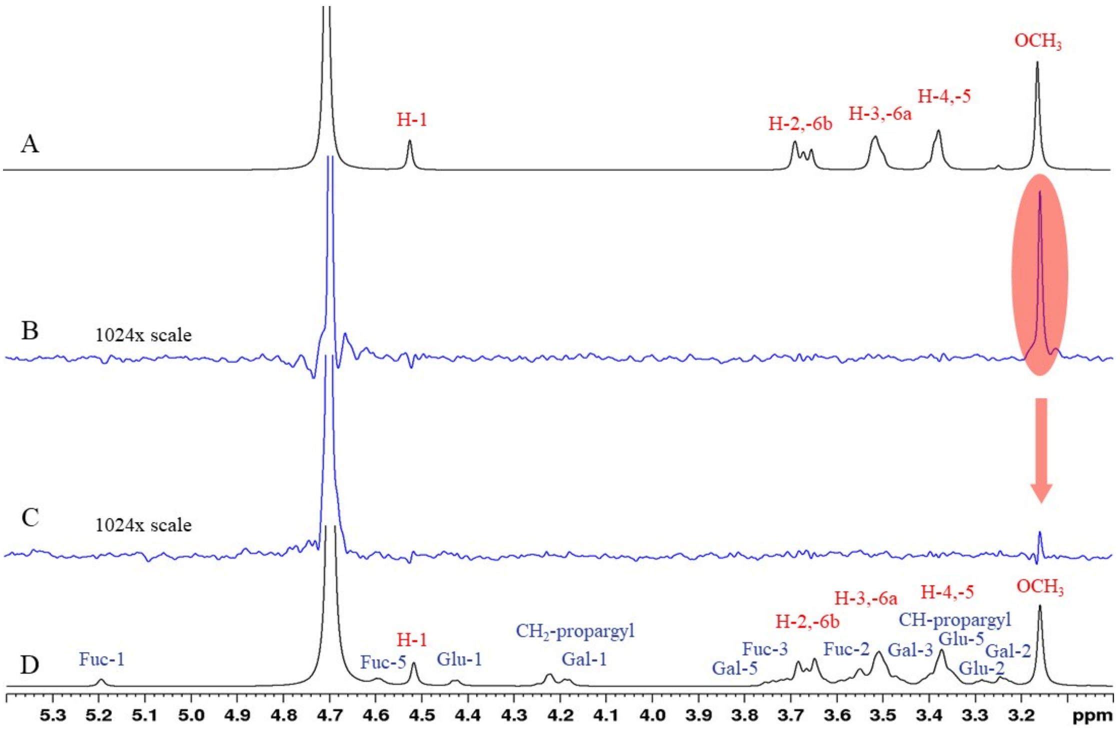

Figure S1. (A) ^{1}H and **(B)** STD NMR spectra of 4 mM Me*α*-D-Man in the presence of 40 μM PA-IIL monomer in D_2O (700 MHz, 20 mM Tris-d_{11}, 150 mM NaCl, 5 mM $CaCl_2$, pH = 7.5, T = 283 K). **(C)** STD and **(D)** ^{1}H spectra of 40 μM PA-IL and 4 mM Me*α*-D-Man titrated propargyl glycoside of 3-*O*-fucosyl lactose **(**Compound **6).** The proton signals of the reference ligand are assigned with red color, while those of the competitor are indicated in blue.

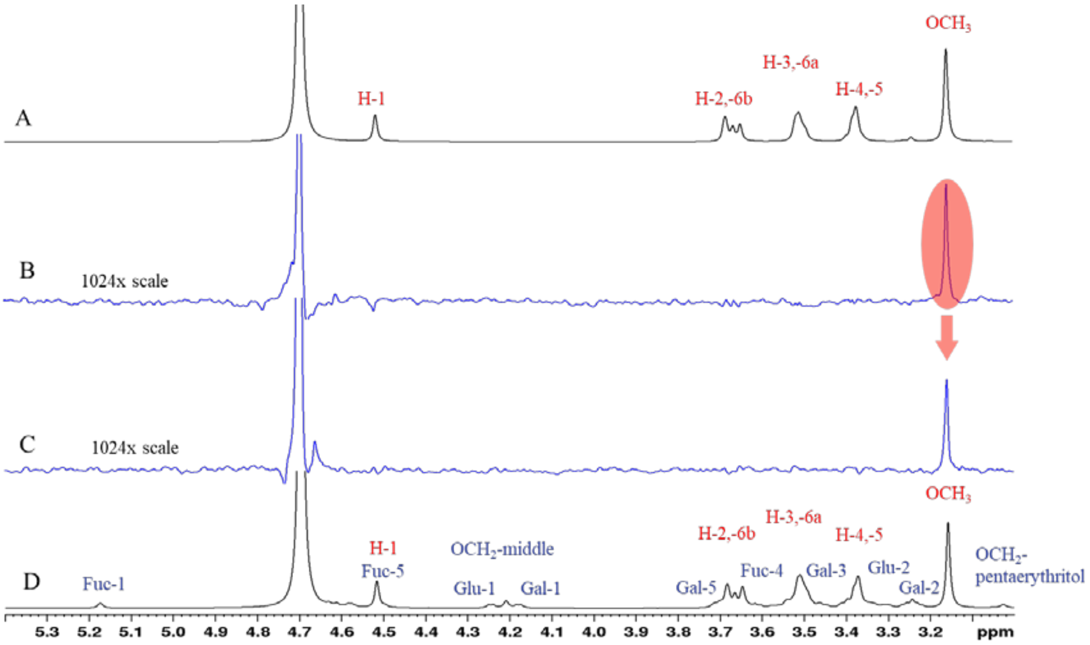

Figure S2. (A) ^{1}H- and **(B)** STD NMR spectra of 4 mM Meα-D-Man in the presence of 40 μM PA-IIL monomer in D_2O (700 MHz, 20 mM Tris-d_{11}, 150 mM NaCl, 5 mM $CaCl_2$, pH = 7.5, T = 283 K). **(C)** STD and **(D)** ^{1}H-NMR spectra of 40 μM PA-IIL and 4 mM Meα-D-Man titrated with 0.4 mM tetravalent 3-fucosyl lactose derivative **(**Compound **8).** The proton signals of the reference ligand are assigned with red color, while those of the competitor are indicated in blue.

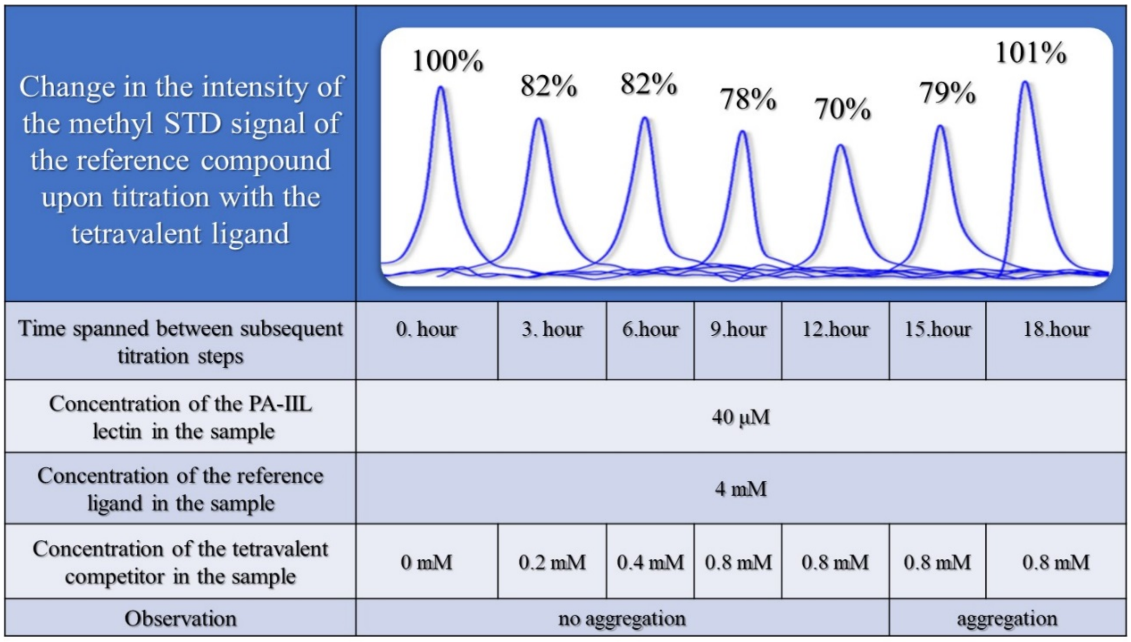

Change in the intensity of the methyl STD signal of the reference compound upon titration with the tetravalent ligand							
Time spanned between subsequent titration steps	0. hour	3. hour	6.hour	9.hour	12.hour	15.hour	18.hour
Concentration of the PA-IIL lectin in the sample	40 μM						
Concentration of the reference ligand in the sample	4 mM						
Concentration of the tetravalent competitor in the sample	0 mM	0.2 mM	0.4 mM	0.8 mM	0.8 mM	0.8 mM	0.8 mM
Observation	no aggregation					aggregation	

Table S1. Intensity change of the methyl STD signal of the reference compound upon titration with the tetravalent ligand **8**.

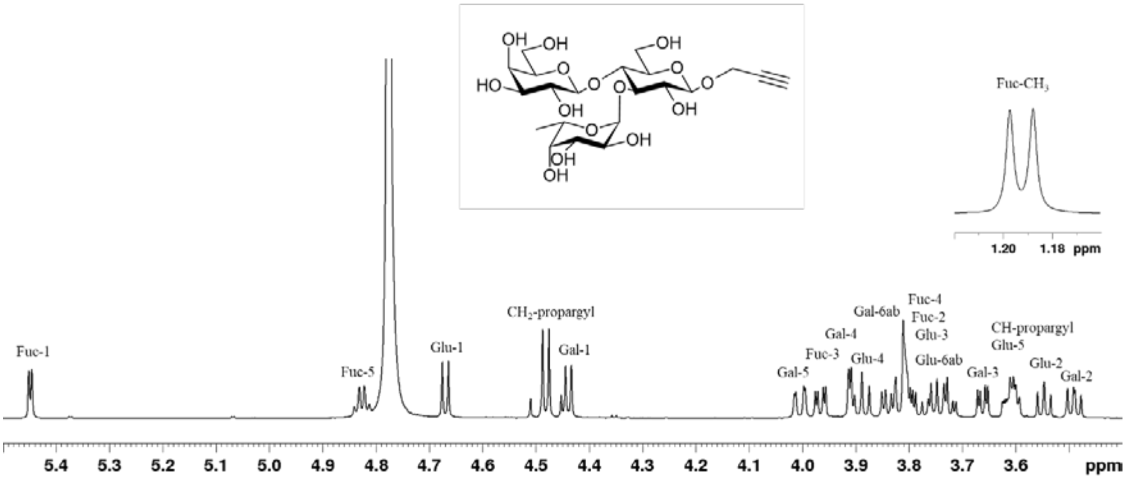

Figure S3. Structure and ^{1}H-NMR spectrum of 5 mM propargyl glycoside of 3-fucosyl lactose (Compound **6**) in D_2O (700 MHz, 20 mM Tris-d_{11}, 150 mM NaCl, 5 mM $CaCl_2$, pH = 7.5, T = 298 K).

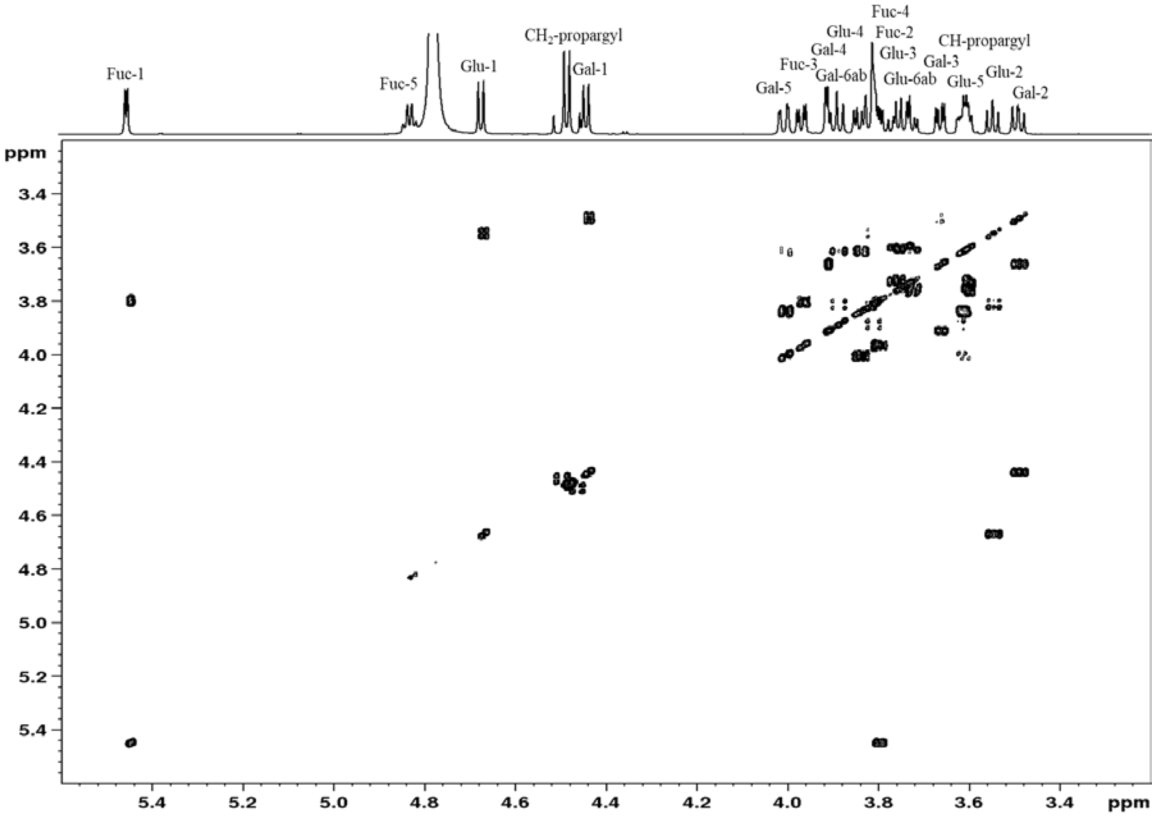

Figure S4. 2D COSY spectrum of 5 mM propargyl glycoside of 3-fucosyl lactose (Compound **6**) in D_2O (700 MHz, 20 mM Tris-d_{11}, 150 mM NaCl, 5 mM $CaCl_2$, pH = 7.5, T = 298 K).

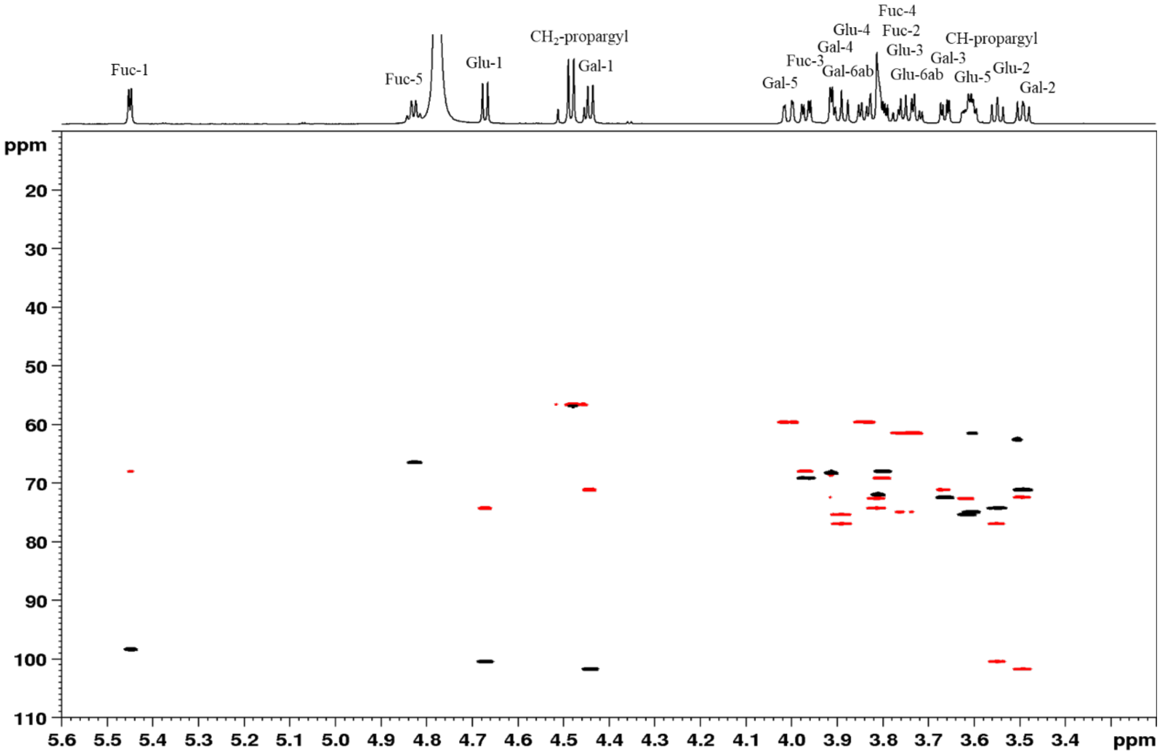

Figure S5. 2D HSQC-CLIP-COSY [1, 2] spectrum of 5 mM propargyl glycoside of 3-fucosyl lactose (Compound **6)** in D_2O (700 MHz, 20 mM Tris-d_{11}, 150 mM NaCl, 5 mM $CaCl_2$, pH = 7.5, T = 298 K). The HSQC peaks are shown in black, while the COSY-type peaks are red.

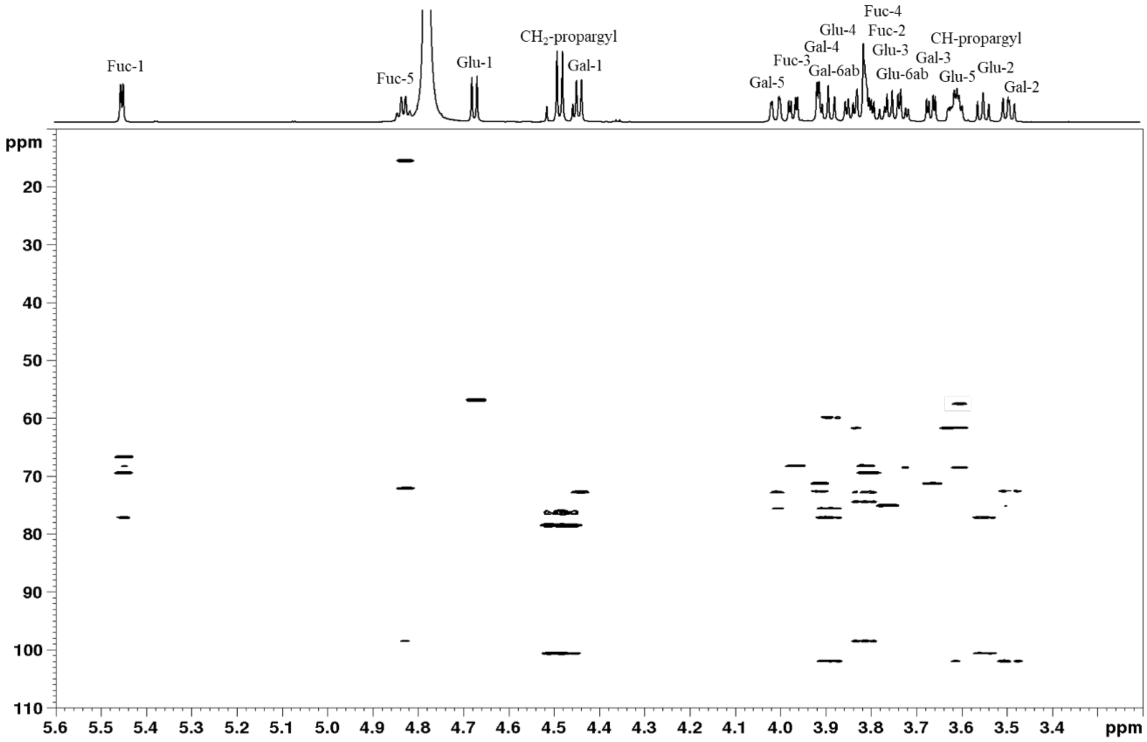

Figure S6. 2D ^1H-^{13}C HMBC spectrum of 5 mM propargyl glycoside of 3-fucosyl lactose (Compound **6)** in D_2O (700 MHz, 20 mM Tris-d_{11}, 150 mM NaCl, 5 mM $CaCl_2$, pH = 7.5, T = 298 K).

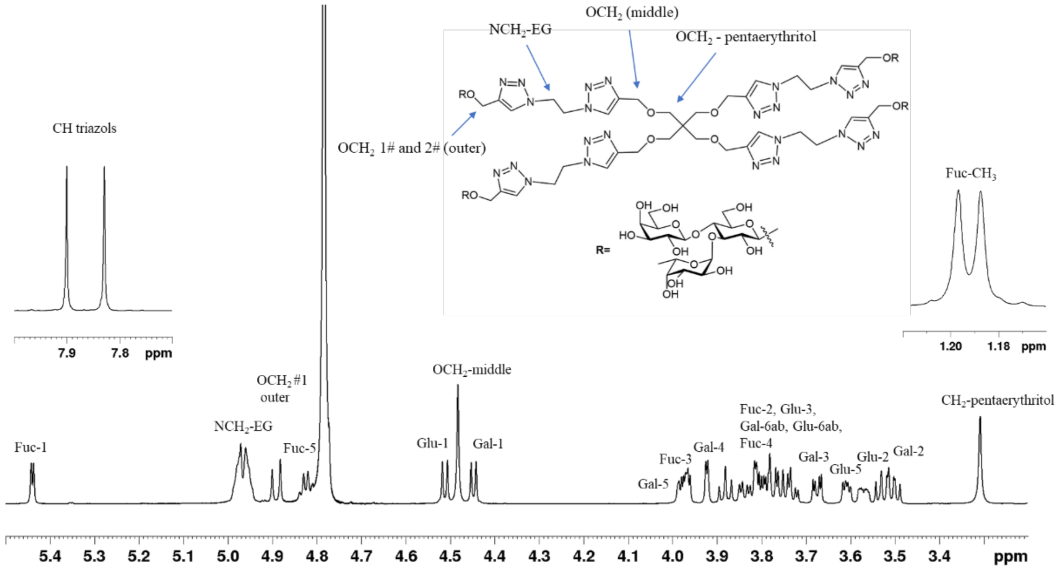

Figure S7. Structure and ¹H-NMR spectrum of 1 mM tetravalent 3-fucosyl lactose derivative (Compound **8)** in D_2O (700 MHz, 20 mM Tris-d_{11}, 150 mM NaCl, 5 mM $CaCl_2$, pH = 7.5, T = 298 K).

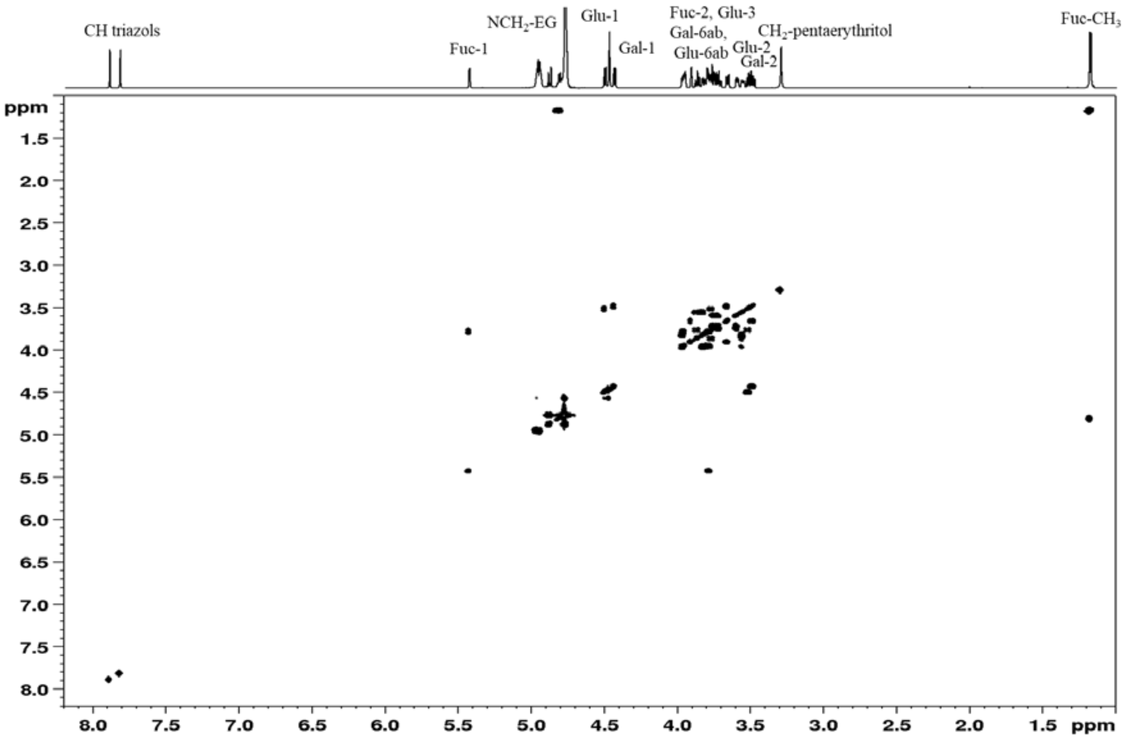

Figure S8. 2D COSY spectrum of 1 mM tetravalent 3-fucosyl lactose derivative (Compound **8)** in D_2O (700 MHz, 20 mM Tris-d_{11}, 150 mM NaCl, 5 mM $CaCl_2$, pH = 7.5, T = 298 K).

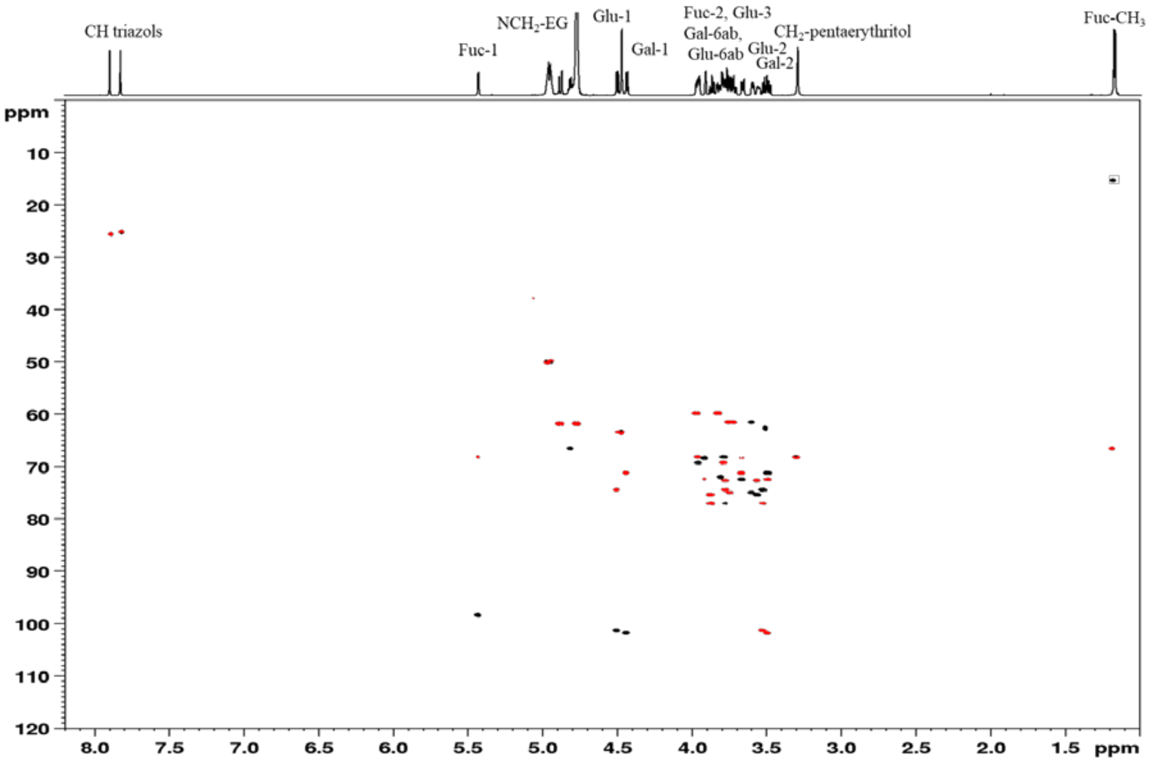

Figure S9. 2D HSQC-CLIP-COSY spectrum of 1 mM tetravalent 3-fucosyl lactose derivative **(**Compound **8)** in D_2O (700 MHz, 20 mM Tris-d_{11}, 150 mM NaCl, 5 mM $CaCl_2$, pH = 7.5, T = 298 K). The HSQC peaks are shown in black, while the COSY-type peaks are red.

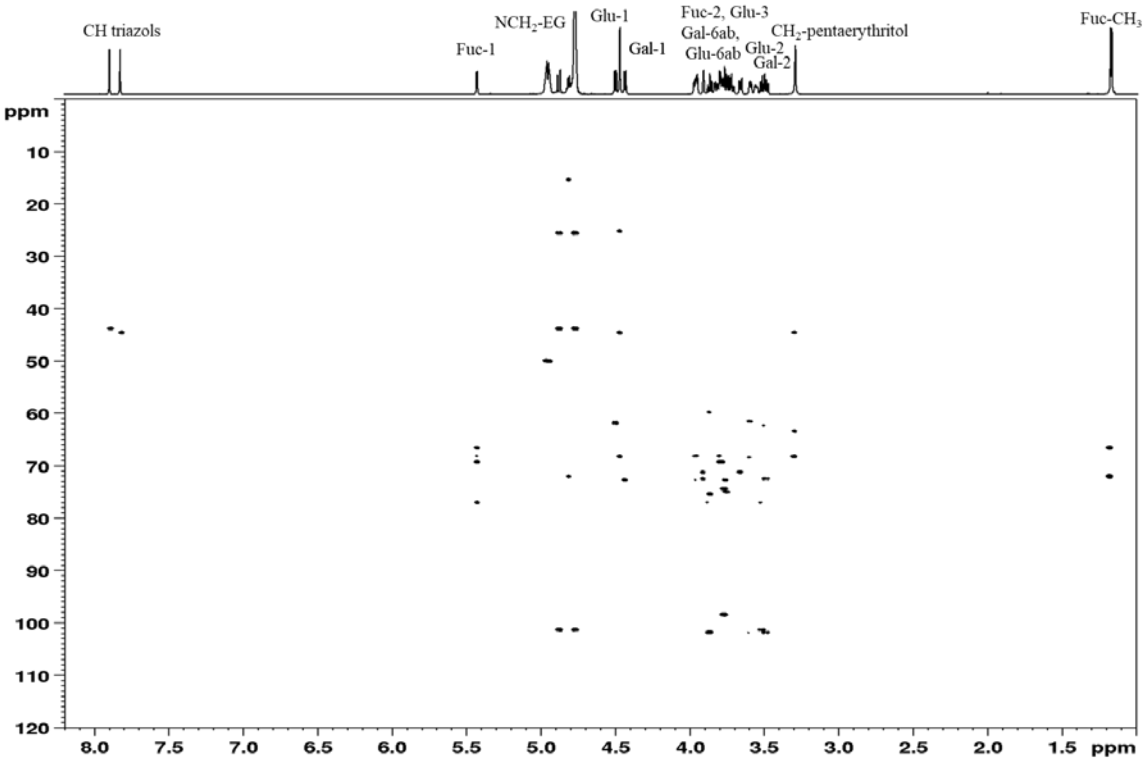

Figure S10. 2D ^{1}H-^{13}C HMBC NMR spectrum of 1 mM tetravalent 3-fucosyl lactose derivative **(**Compound **8)** in D_2O (700 MHz, 20 mM Tris-d_{11}, 150 mM NaCl, 5 mM $CaCl_2$, pH = 7.5, T = 298 K).

References

1. Gyöngyösi, T., Timári, I., Sinnaeve, D., Luy B., Kövér, E. K. Expedited Nuclear Magnetic Resonance Assignment of Small- to Medium-Sized Molecules with Improved HSQC-CLIP-COSY Experiments. *Anal. Chem.*, **2021**, *93*, 3096-3102. doi: 10.1021/acs.analchem.0c04124.

2. Gyöngyösi, T., Timári, I., Haller, J., Koos, M. R. M., Luy, B., Kövér, K. E. Boosting the NMR Assignment of Carbohydrates with Clean In-Phase Correlation Experiments. *ChemPlusChem*, **2018**, *83*, 53-60. doi: 10.1002/cplu.201700452
